# Post-intensive care syndrome and health-related quality of life in long-term survivors of cardiac arrest: a prospective cohort study

**DOI:** 10.1038/s41598-024-61146-8

**Published:** 2024-05-08

**Authors:** Simon A. Amacher, Christian Sahmer, Christoph Becker, Sebastian Gross, Armon Arpagaus, Tabita Urben, Kai Tisljar, Christian Emsden, Raoul Sutter, Stephan Marsch, Sabina Hunziker

**Affiliations:** 1 Intensive Care Medicine, Department of Acute Medical Care, University Hospital Basel, Basel, Switzerland; 2 Medical Communication and Psychosomatic Medicine, University Hospital Basel, Klingelbergstrasse 23, 4031 Basel, Switzerland; 3 Emergency Medicine, Department of Acute Medical Care, University Hospital Basel, Basel, Switzerland; 4 Post-Intensive Care Clinic, University Hospital Basel, Basel, Switzerland; 5 Medical Faculty, University of Basel, Basel, Switzerland; 6 Division of Neurophysiology, Department of Neurology, University Hospital Basel, Basel, Switzerland

**Keywords:** Cardiopulmonary resuscitation, Long-term outcomes, Post-intensive care syndrome, Outcomes research, Cardiology

## Abstract

Patients discharged from intensive care are at risk for post-intensive care syndrome (PICS), which consists of physical, psychological, and/or neurological impairments. This study aimed to analyze PICS at 24 months follow-up, to identify potential risk factors for PICS, and to assess health-related quality of life in a long-term cohort of adult cardiac arrest survivors. This prospective cohort study included adult cardiac arrest survivors admitted to the intensive care unit of a Swiss tertiary academic medical center. The primary endpoint was the prevalence of PICS at 24 months follow-up, defined as impairments in physical (measured through the European Quality of Life 5-Dimensions-3-Levels instrument [EQ-5D-3L]), neurological (defined as Cerebral Performance Category Score > 2 or Modified Rankin Score > 3), and psychological (based on the Hospital Anxiety and Depression Scale and the Impact of Event Scale-Revised) domains. Among 107 cardiac arrest survivors that completed the 2-year follow-up, 46 patients (43.0%) had symptoms of PICS, with 41 patients (38.7%) experiencing symptoms in the physical domain, 16 patients (15.4%) in the psychological domain, and 3 patients (2.8%) in the neurological domain. Key predictors for PICS in multivariate analyses were female sex (adjusted odds ratio [aOR] 3.17, 95% CI 1.08 to 9.3), duration of no-flow interval during cardiac arrest (minutes) (aOR 1.17, 95% CI 1.02 to 1.33), post-discharge job-loss (aOR 31.25, 95% CI 3.63 to 268.83), need for ongoing psychological support (aOR 3.64, 95% CI 1.29 to 10.29) or psychopharmacologic treatment (aOR 9.49, 95% CI 1.9 to 47.3), and EQ-visual analogue scale (points) (aOR 0.88, 95% CI 0.84 to 0.93). More than one-third of cardiac arrest survivors experience symptoms of PICS 2 years after resuscitation, with the highest impairment observed in the physical and psychological domains. However, long-term survivors of cardiac arrest report intact health-related quality of life when compared to the general population. Future research should focus on appropriate prevention, screening, and treatment strategies for PICS in cardiac arrest patients.

## Background

Sudden cardiac arrest is a leading global health issue, with survivors frequently experiencing physical, neurological, and psychological sequelae^1–4^. Approximately 18 to 35% of patients experiencing an in-hospital cardiac arrest (IHCA) survive to hospital discharge, whereas the survival rates for out-of-hospital cardiac arrest (OHCA) are substantially lower, with 9 to 10% surviving to hospital discharge^2,5^. In Europe, approximately 90% of survivors to hospital discharge experience a good neurological outcome as defined by a cerebral performance category (CPC) score of ≤ 2^5^. In recent years, the focus of the critical care community has shifted from acute illness towards an emphasis on long-term sequelae after discharge from the intensive care unit^6,7^. Hence, in 2012, a North American critical care stakeholder conference agreed upon the term post-intensive care syndrome (PICS), which is defined as a new or worsening physical, mental, or neurocognitive disorder affecting patients’ quality of life after discharge from intensive care^8^. Multiple cohort studies have found evidence of high rates of PICS in ICU survivors. This is true for general intensive care unit (ICU) ^9^ patients and special populations such as treatment with extracorporeal membrane oxygenation, severe Coronavirus disease—2019 infections, acute respiratory distress syndrome, or subarachnoid hemorrhage^4,10–15^. PICS is an important driver of premature mortality and can have substantial financial implications for patients and their families^16–18^. Physical, psychological, and cognitive long-term disabilities are well-known in cardiac arrest survivors^4,19–25^. In a previous study, our research group found evidence of PICS in 50% of survivors at 12 months of follow-up, with most impairments experienced in the physical domain^4^. However, studies looking at the concept of PICS and health-related quality of life in cardiac arrest patients surviving longer than 1 year are very scarce^4^. The present study aimed to assess the prevalence of PICS at a 24-month follow-up, to identify potential risk factors for PICS, and to assess health-related quality of life in a well-defined long-term cohort of adult cardiac arrest survivors.

## Materials and methods

### Study setting

The COMMUNICATE/PROPHETIC study is an ongoing prospective cohort study and includes consecutive adult cardiac arrest patients admitted to a 42-bed interdisciplinary intensive care unit of a tertiary teaching hospital (University Hospital Basel, Switzerland). The study aimed to identify risk factors and prognostic markers for neurological outcomes after cardiac arrest with a maximum follow-up of 24 months. Details of the study’s conduct and procedures have been published previously^4,26–33^. Patients were treated in accordance with the local treatment protocol, which followed the respective recommendations from the European Resuscitation Council^34–36^. The data collection, analysis, and reporting followed the Strengthening the Reporting of Observational Studies in Epidemiology (STROBE) guidelines^37^.

### Ethical approval

The study was approved by the local ethics committee (Ethics Committee of Northwest and Central Switzerland, www.eknz.ch, Ref. No. 2019-01162) and complies with the declaration of Helsinki and its amendments^38^. Informed consent was obtained from all subjects and/or their legal guardian(s).

### Participants

All consecutive adult patients (i.e., ≥ 18 years of age) with a return of spontaneous circulation (ROSC) admitted to the ICU after an out-of-hospital or non-monitored in-hospital cardiac arrest were prospectively included from October 2012 to December 2020 in the COMMUNICATE/PROPHETIC cohort. All patients who completed the 24-month follow-up were included in the present analysis.

### Data collection and follow-up

The study team prospectively collected data upon ICU admission from the digital ICU’s patient data management system and the hospital medical record. Patients who survived to hospital discharge were contacted 3 months, 12 months, and 24 months after discharge to perform a predefined and structured telephone interview, lasting for about 20 minutes. The telephone interviews were performed by psychologists with a master’s degree in psychology or physicians. Patients needing further support were referred to the respective outpatient clinic.

### Measures

#### Baseline and predictor variables

The patient’s sociodemographic variables (e.g., age, sex, job status on admission) and comorbidities (e.g., arterial hypertension, diabetes), length of hospital and ICU stay, and duration of post-discharge inpatient rehabilitation were extracted from the hospital record. The job status was updated at each follow-up visit. Additionally, data regarding the cardiac arrest characteristics (e.g., location, initial rhythm, no-flow time, low-flow time), presumed cardiac arrest etiology (e.g., acute coronary syndrome, arrhythmogenic, other), ICU treatment and interventions (e.g., intubation, targeted temperature management, vasoactive drugs), ICU complications (e.g., delirium, pneumonia) was collected. No-flow time was defined as the time from the onset of cardiac arrest until the beginning of basic life support measures. Low-flow time was defined as the time from the beginning of basic life support measures until the return of spontaneous circulation (ROSC) in accordance with the literature^39^. The sum of no-flow and low-flow time was defined as time until ROSC. Serum concentrations of neuron-specific enolase (as micrograms per liter) were assessed at 48 and 72 h according to recent European Resuscitation Council guidelines^34^. Patient characteristics, including comorbidities and clinical parameters at ICU admission, were used to calculate the severity of illness scores Acute Physiology and Chronic Health Disease Classification System (APACHE) II score and Simplified Acute Physiology Score (SAPS) II according to the original publications^40,41^.

#### Primary outcome

The primary outcome was defined as the prevalence of PICS at 24 months of follow-up. According to recent literature in the field ^42^, PICS was defined as symptoms or impairment in at least one of the subsequent domains: Physical, psychological, and/or neurological.

Physical impairment was assessed using the European Quality of Life 5-dimensions, 3-levels instrument (EQ-5D-3L), an extensively validated self-report tool assessing the general quality of life in five dimensions (mobility, self-care, usual activities, pain/discomfort, anxiety/depression^43,44^. For each dimension, there are three levels of possible responses: *Level 1*—no problems; *Level 2*—some problems; *Level 3*—extreme problems/unable to^44^. Physical impairment was coded as present if symptoms or impairment in at least one of the “mobility”-, “self-care”- and/or “usual activities” dimensions was reported.

Psychological impairment was defined as experiencing symptoms of depression, anxiety and/or post-traumatic stress disorder (PTSD).

The Hospital Anxiety and Depression Scale (HADS), a self-report instrument explicitly designed for hospitalized patients with medical conditions, was utilized to evaluate symptoms of anxiety and depression^45^. The HADS is reliable and well-validated when utilizing a cutoff score of ≥ 8 on the depression and/or anxiety subscale; an optimal balance between sensitivity and specificity can be achieved^46^. Hence, a cutoff of ≥ 8 on the anxiety and/or depression subscale was used as evidence of clinically relevant symptoms in the domains of anxiety and depression within the context of this study.

For the assessment of PTSD symptoms, the Impact of Event Scale-Revised (IES-R) was used^47^. The IESR is a well-validated and reliable self-report instrument with 22 items subdivided into the three symptom complexes of PTSD: Intrusion, avoidance, and hyperarousal^47^. In the development study, the German version of IES-R showed good diagnostic accuracy for PTSD at a cutoff of 0^48^. Hence, the same cutoff was used within the context of this study^48^.

*Neurological impairment* was approximated using the cerebral performance category scale (CPC), a well-validated expert-rated scale with good inter-rater reliability for assessing neurological outcomes after cardiac arrest^49,50^. According to the original publication^50^, the CPC classifies neurological outcomes after brain damage into five categories ranging from good recovery and resumption of normal life to death, including brain death (Box 1).

**Box 1:** Cerebral performance category scale (CPC) categories. Adapted from Jennett et al. Lancet. 1975;1(7905):480–4.

CPC 1	“Good recovery—(…) Resumption of normal life even though there may be minor neurological and psychological deficits”
CPC 2	“Moderate disability—Disabled but independent in activities of daily living, such as the use of public transportation or doing groceries.”
CPC 3	“Severe disability—conscious but disabled. (…) Patients dependent for daily support by reason of mental or physical disability, usually a combination of both”
CPC 4	“Persistent vegetative state”
CPC 5	“Death”, including brain death

In accordance with expert consensus statements, the neurological outcome was dichotomized into good neurological outcome (CPC 1–2) and bad neurological outcome (CPC 3–5)^51,52^.

#### Secondary outcomes

The key secondary outcome was the health-related quality of life assessed at 24 months of follow-up as measured by EQ-5D-3L and the European Quality of Life-visual analogue scale (EQ-VAS). The EQ-5D-3L is described above. For the EQ-VAS, the patients were asked to self-rate their general state of health on the day of follow-up on a scale from 0 to 100, mimicking a thermometer. On the scale, 0 indicates the worst health condition- and 100 the best imaginable health condition^44^. The EQ-VAS was previously validated independently from the complete EQ-5D-3L as a measure for health-related quality of life^53^. The EQ-5D-3L, and the EQ-VAS results were then compared to the population norms of a representative sample of the Swiss population^54^. Additionally, the EQ-VAS was dichotomized along the 25th percentile of the EQ-VAS (lower quartile versus upper quartiles).

### Statistical analysis

The study population’s sociodemographic characteristics, comorbidities, and clinical characteristics were analyzed by descriptive statistics, i.e., frequencies/percentages for categorical variables; medians and interquartile ranges (IQR) for non-normal distributions of continuous or discrete data. We analyzed potential predictors for the primary endpoint, i.e., patients with PICS at 24 months of follow-up, using univariate logistic regression. Additionally, we adjusted the analyses for age and sex. Odds ratios (OR) with 95% confidence intervals (95% CI) were reported as measures of association. Missing predictor values were imputed by chained equations using multiple covariables (i.e. sociodemographics, comorbidities) and main outcomes (prevalence of PICS, death, neurological outcome) as previously suggested by Sterne et al.^55^ All statistical analyzes were performed with the STATA 15.0 (StataCorp., College Station, Texas, United States of America) software.

## Results

### Study population and baseline characteristics

**Figure 1:**
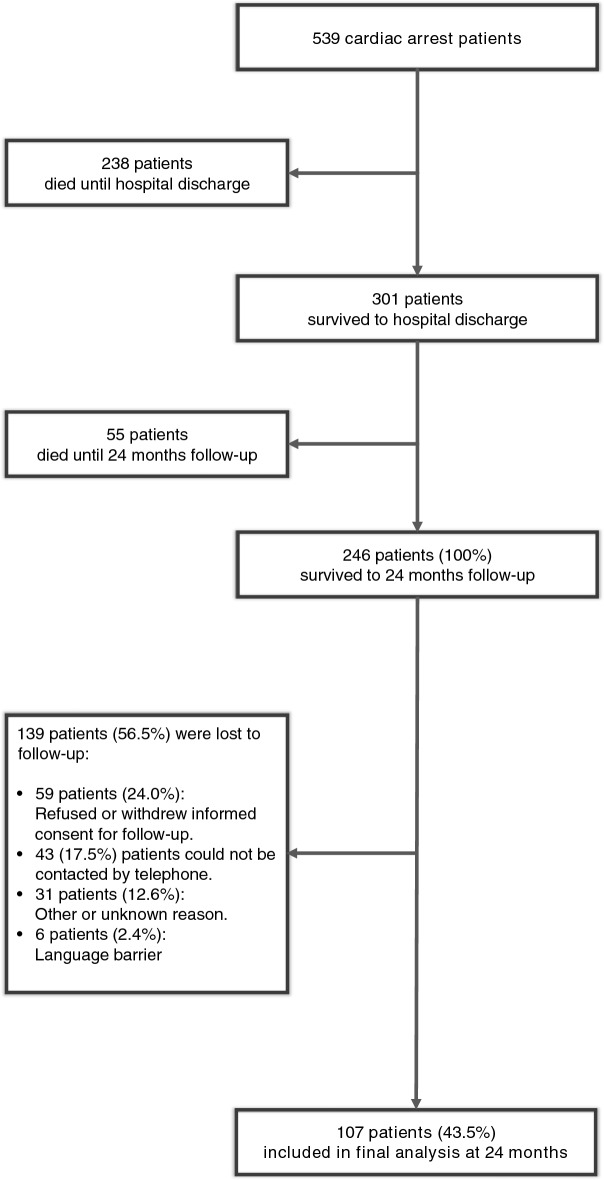
Flow chart of the study population.

Of 246 patients alive 24 months after cardiac arrest, 139 patients were lost to follow-up, resulting in 107 patients that completed the 24-month follow-up and were finally included in the present analysis. However, when comparing the baseline characteristics and important in-hospital outcomes (i.e., neurological outcome at hospital discharge), the lost-to-follow-up patients did not differ from those included in the final analysis (Supplementary Table 2). Figure 1 displays the study flow chart. Details of the study population’s baseline characteristics are shown in Table 1.

**Table 1:** Baseline characteristics of study population.

Sociodemographics	
N	107
Age, median (IQR)	62.4 (54.3, 71.9)
Female sex, n (%)	18 (16.8)
Relationship, n (%)	88 (83.0)
Children, n (%)	88 (82.2)
Highest education	
School, n (%)	9 (8.4)
Diploma/apprenticeship, n (%)	71 (66.4)
University, n (%)	17 (15.9)
Unknown, n (%)	10 (9.3)
Employed at baseline, n (%)	57 (54.8)
Comorbidities	
Coronary heart disease, n (%)	74 (69.2)
Heart failure, n (%)	8 (7.5)
COPD, n (%)	5 (4.7)
Liver cirrhosis, n (%)	3 (2.8)
Arterial hypertension, n (%)	53 (49.5)
Diabetes, n (%)	13 (12.1)
Chronic kidney disease, n (%)	6 (5.6)
Neurological disease, n (%)	7 (6.5)
Severity of illness scores at ICU admission	
APACHE II score, median (IQR)	27.5 (23, 31)
SAPS II score, median (IQR)	60 (47, 67)
Cardiac arrest characteristics	
Etiology: Acute coronary syndrome, n (%)	76 (72.4)
Etiology: Rhythmogenic, n (%)	17 (16.2)
Etiology: Other or unknown reason, n (%)	12 (11.4)
Setting of cardiac arrest	
At home, n (%)	35 (33.0)
In public, n (%)	60 (56.6)
IHCA, n (%)	11 (10.4)
Observed cardiac arrest, n (%)	99 (92.5)
Bystander CPR, n (%)	86 (80.4)
Professional bystander CPR, n (%)	29 (52)
Initial rhythm	
VT, n (%)	6 (5.6)
VF, n (%)	80 (74.8)
Asystolie, n (%)	2 (1.9)
PEA, n (%)	6 (5.6)
Unknown, n (%)	13 (12.1)
No-flow (min), median (IQR)	.5 (.5, 2)
Low-flow (min), median (IQR)	13 (8, 20)
Time until ROSC, median (IQR)	15 (10, 26)
Epinephrine during resuscitation	
No epinephrine, n (%)	54 (55)
< 3 mg, n (%)	20 (20)
≥ 3 mg, n (%)	24 (24)

APACHE II, acute physiology and chronic health evaluation score II; COPD, Chronic obstructive pulmonary disease; CPR, Cardiopulmonary resuscitation; IHCA In-hospital cardiac arrest; IQR Interquartile range; ROSC Return of spontaneous circulation; VF, ventricular fibrillation; VT, pulseless ventricular tachycardia; PEA, pulseless electrical activity; SAPS II, simplified acute physiology score II.

### Primary outcome: prevalence of PICS 24 months after cardiac arrest

Among the 107 included patients, 46 (43.0%) had evidence of PICS, with 41 patients (38.7%) experiencing symptoms in the physical domain, 16 patients (15.4%) in the psychological domain, and 3 patients (2.8%) in the neurological domain.

In particular, 41 patients (38.7%) had physical impairment in the EQ-5D-3L, with 26 patients (24.5%) reporting ‘mobility problems, 7 patients (6.6%) ‘self-care’ problems, and 30 patients (28.3%) having problems with ‘usual activities’.

Five patients (5%) presented with signs of PTSD in the IES-R. Measured by the HADS, 12 patients (11.9%) had symptoms of anxiety, and 10 patients (9.7%) had evidence of depression as an expression of psychological sequelae.

Finally, 3 patients (2.8%) had significant neurological impairment, as evidenced by a clinician-reported score of 3 or 4 on the CPC. This rating scale does not allow a finer-grained evaluation of cognitive status. Therefore, the number of patients with ongoing cognitive impairment might be underestimated.

Twelve patients (11.2%) had evidence of impairment in the physical and psychological domain, 2 patients (1.9%) had evidence of PICS in the physical and neurological domain, and no patient had evidence of PICS in the psychological and neurological domain or in all domains, respectively.

When looking at the subgroup of patients with impairments in the physical domain, these patients were significantly more often female (11 [27%] vs. 7 [11%], *p* = 0.032) and more often needed mechanical circulatory support during the ICU stay (9 [22%] vs. 1 [2%], *p* < 0.001) (Supplementary Table 3).

The association of several potential predictors for PICS adjusted for age and sex was assessed. Thereby, several key predictors for PICS were identified: Female sex (adjusted OR 3.17, 95% CI 1.08 to 9.3; *p* = 0.036), no-flow time (adjusted OR 1.17, 95% CI 1.02 to 1.33, *p* = 0.026), post-discharge job loss (adjusted OR 31.25, 95% CI 3.63 to 268.83; *p* = 0.002), ongoing need for psychological support (adjusted OR 3.64, 95% CI 1.29 to 10.29; *p* = 0.015), and ongoing need for psychopharmacologic treatment (adjusted OR 9.49, 95% CI 1.9 to 47.3; *p* = 0.006), EQ-VAS (adjusted OR 0.88, 95% CI 0.84 to 0.93; *p* < 0.001). Details regarding further predictors can be obtained from Tables 2 and 3.

**Table 2:** Predictors for PICS at 24 months of follow-up.

Sociodemographics	No PICS	PICS	OR (95% CI)	*p*-value	adjusted OR* (95% CI)	*p*-value
N	61	46				
Age (years), median (IQR)	63.5 (57.4, 72.4)	61.2 (52.5, 70.2)	0.99 (0.96, 1.02)	0.548	0.99 (0.96, 1.02)	0.548
Female sex, n (%)	6 (10)	12 (26)	3.24 (1.11, 9.42)	**0.031**	3.17 (1.08, 9.3)	**0.036**
Relationship, n (%)	52 (85)	36 (80)	0.69 (0.25, 1.91)	0.479	0.76 (0.27, 2.17)	0.611
Children, n (%)	51 (84)	37 (80)	0.81 (0.3, 2.18)	0.671	0.8 (0.29, 2.25)	0.677
Highest education						
School, n (%)	4 (7)	5 (11)	0.16 (0.02, 1.37)	0.094	0.15 (0.02, 1.33)	0.088
Diploma/apprenticeship, n (%)	39 (64)	32 (70)	1.85 (0.71, 4.8)	0.208	1.22 (0.52, 2.82)	0.649
University, n (%)	14 (23)	3 (7)	0.25 (0.07, 0.93)	0.039	0.67 (0.26, 1.73)	0.408
Employed at baseline, n (%)	32 (54)	25 (56)	1.05 (0.48, 2.3)	0.894	1.13 (0.41, 3.08)	0.814
Comorbidities						
Coronary heart disease, n (%)	42 (69)	32 (70)	1.03 (0.45, 2.37)	0.937	1.37 (0.56, 3.35)	0.495
Heart failure, n (%)	6 (10)	2 (4)	0.42 (0.08, 2.17)	0.298	0.52 (0.1, 2.74)	0.439
COPD, n (%)	2 (3)	3 (7)	2.06 (0.33, 12.85)	0.44	2.66 (0.42, 16.95)	0.301
Liver cirrhosis, n (%)	2 (3)	1 (2)	0.66 (0.06, 7.46)	0.734	0.82 (0.07, 9.46)	0.875
Arterial hypertension, n (%)	30 (49)	23 (50)	1.03 (0.48, 2.22)	0.933	1.06 (0.48, 2.38)	0.878
Diabetes, n (%)	7 (11)	6 (13)	1.16 (0.36, 3.71)	0.806	1.02 (0.29, 3.66)	0.974
Chronic kidney disease, n (%)	4 (7)	2 (4)	0.65 (0.11, 3.7)	0.625	0.64 (0.11, 3.83)	0.625
Neurological disease, n (%)	3 (5)	4 (9)	1.84 (0.39, 8.66)	0.44	1.81 (0.35, 9.47)	0.48
Cardiac arrest characteristics						
Etiology						
Acute coronary syndrome, n (%)	43 (73)	33 (72)	0.94 (0.4, 2.23)	0.897	1.18 (0.47, 3)	0.724
Rhythmogenic, n (%)	10 (17)	7 (15)	0.88 (0.31, 2.52)	0.811	0.8 (0.27, 2.41)	0.694
Other reason or unknown, n (%)	6 (10)	6 (13)	1.33 (0.4, 4.42)	0.647	0.98 (0.27, 3.57)	0.977
Setting of cardiac arrest						
At home, n (%)	19 (31)	16 (36)	(Ref)		(Ref)	
In public, n (%)	39 (64)	21 (47)	0.64 (0.27, 1.50)	0.30	0.77 (0.32, 1.87)	0.56
IHCA, n (%)	3 (5)	8 (18)	3.17 (0.72, 13.99)	0.71	3.40 (0.74, 15.58	0.12
Observed cardiac arrest, n (%)	54 (89)	45 (98)	5.83 (0.69, 49.2)	0.105	4.76 (0.56, 40.64)	0.153
Bystander CPR, n (%)	50 (82)	36 (78)	0.79 (0.3, 2.06)	0.633	0.71 (0.26, 1.96)	0.514
Professional bystander CPR, n (%)	19 (54)	10 (48)	0.77 (0.26, 2.26)	0.629	0.74 (0.22, 2.45)	0.622
Initial rhythm						
VT, n (%)	4 (7)	2 (4)	(Ref)		(Ref)	
VF, n (%)	42 (69)	38 (83)	1.81 (0.31, 10.44)	0.50	1.89 (0.30, 11.70)	0.49
Asystolie, n (%)	2 (3)	0 (0)	NA	NA	NA	NA
PEA, n (%)	3 (5)	3 (7)	NA	NA	NA	NA
Unknown, n (%)	10 (16)	3 (7)	NA	NA	NA	NA
Resuscitation parameters						
No-flow (min), median (IQR)	.5 (.5, 2)	.5 (.5, 5)	1.15 (1.01, 1.33)	**0.042**	1.17 (1.02, 1.33)	**0.026**
Low-flow (min), median (IQR)	11 (8, 20)	14 (9, 30)	1.02 (0.99, 1.06)	0.15	1.03 (1, 1.06)	0.091
Time until ROSC (min), median (IQR)	15 (9, 20)	19.5 (10, 30)	1.03 (1, 1.06)	0.076	1.04 (1, 1.07)	**0.033**
Epinephrine during CPR						
No epinephrine, n (%)	37 (65%)	17 (41)	(Ref)		(Ref)	
< 3 mg, n (%)	11 (19%)	9 (22)	1.78 (0.62, 5.10)	0.28	1.96 (0.63, 6.03)	0.24
≥ 3 mg, n (%)	9 (16%)	15 (37)	3.62 (1.32, 9.92)	0.01	3.92 (1.38, 11.02)	**0.01**
Clinical scores at ICU admission						
Glasgow Coma Scale, median (IQR)	3 (3, 13)	3 (3, 14)	0.99 (0.92, 1.07)	0.869	0.99 (0.91, 1.07)	0.788
APACHE II score, median (IQR)	27 (22.5, 30.5)	28 (23, 31)	1.02 (0.96, 1.07)	0.571	1.03 (0.98, 1.08)	0.287
SAPS II score, median (IQR)	59 (41, 67)	60 (52, 66)	1.01 (0.98, 1.04)	0.397	1.01 (0.99, 1.04)	0.354
ICU parameters						
pH, median (IQR)	7.28 (7.22, 7.33)	7.31 (7.21, 7.36)	3.16 (0.03, 289.13)	0.618	2.15 (0.03, 153.85)	0.725
Lactate, median (IQR)	4.35 (2.5, 6.35)	4.45 (2.3, 6.9)	1.03 (0.91, 1.16)	0.677	1.04 (0.91, 1.17)	0.588
Potassium (mmol/l), median (IQR)	4.1 (3.8, 4.8)	4.3 (4, 4.8)	1.42 (0.82, 2.46)	0.207	1.62 (0.90, 2.91)	0.106
Intubated at ICU admission, n (%)	43 (70)	35 (76)	1.33 (0.56, 3.19)	0.52	1.26 (0.51, 3.11)	0.611
Duration of invasive ventilation (days), median (IQR)	1 (0, 2)	1 (.5, 3)	1.14 (0.97, 1.35)	0.118	1.16 (0.96, 1.41)	0.124
Targeted Temperature Management, n (%)	33 (54)	25 (54)	1.01 (0.47, 2.18)	0.98	1.06 (0.46, 2.43)	0.891
Sedation, n (%)	52 (85)	42 (91)	1.82 (0.52, 6.32)	0.347	1.8 (0.49, 6.55)	0.372
NSE (ug/l)—day 2, median (IQR)	20.4 (17.4, 25.8)	24.6 (17.3, 31.1)	1.02 (1, 1.05)	0.078	1.03 (1.01, 1.06)	**0.016**
NSE (ug/l)—day 3, median (IQR)	18.6 (16.4, 21.7)	19.4 (16.3, 33.4)	1.04 (1, 1.08)	0.065	1.03 (1.01, 1.05)	**0.013**
ICU length of stay (days), median (IQR)	4 (2, 7)	4 (2, 7)	1.05 (0.97, 1.13)	0.245	1.05 (0.96, 1.14)	0.274
ICU complications						
Aspiration, n (%)	30 (49)	16 (35)	0.55 (0.25, 1.21)	0.138	0.6 (0.27, 1.35)	0.216
Pneumonia, n (%)	32 (52)	19 (41)	0.64 (0.29, 1.38)	0.254	0.71 (0.31, 1.59)	0.401
Major hemorrhage, n (%)	4 (7)	3 (7)	0.99 (0.21, 4.68)	0.994	1.03 (0.21, 5.02)	0.972
Delirium, n (%)	17 (28)	16 (35)	1.38 (0.6, 3.15)	0.444	1.54 (0.66, 3.62)	0.32
Acute Kidney Injury, n (%)	5 (8)	8 (17)	2.36 (0.72, 7.76)	0.158	2.29 (0.68, 7.75)	0.183
Epileptic seizure, n (%)	2 (3)	5 (11)	3.6 (0.67, 19.45)	0.137	4.55 (0.82, 25.42)	0.084

Bold values represent p-values < 0.05.

*OR adjusted for age and/or sex category, respectively.

APACHE II, acute physiology and chronic health evaluation score II; CPC, cerebral performance category; COPD, chronic obstructive pulmonary disease; CPR, cardiopulmonary resuscitation; ICU, intensive care unit; IHCA, in-hospital cardiac arrest; IQR, interquartile range; NA, not applicable due to low sample size; NSE, neurone-specific enolase; OR, odds ratio; PEA, pulseless electrical activity; PICS, post-intensive care syndrome; ROSC, return of spontaneous circulation; SAPS II, simplified acute physiology score II; VF, ventricular fibrillation; VT, pulseless ventricular tachycardia.

**Table 3:** Predictors for PICS at 24 months of follow-up.

	No PICS	PICS	OR (95% CI)	*p*-value	adjusted OR* (95% CI)	*p*-value
Hospital discharge parameters						
Hospital length of stay (days), median (IQR	11 (7, 15)	14 (9, 18)	1.05 (1, 1.1)	0.072	1.05 (0.99, 1.11)	0.078
Poor neurological outcome (CPC 3–5), n (%)	1 (2)	3 (7)	4.19 (0.42, 41.62)	0.222	5.17 (0.5, 53.23)	0.167
Parameters at 24 months of follow-up						
Rehabilitation						
None, n (%)	18 (30)	13 (28)	1.49 (0.28, 7.97)	0.644	0.91 (0.38, 2.19)	0.837
Up to 3 Weeks, n (%)	20 (33)	13 (28)	0.81 (0.35, 1.86)	0.616	0.87 (0.37, 2.04)	0.745
More than 3 Weeks, n (%)	23 (38)	20 (43)	1.27 (0.58, 2.77)	0.547	1.23 (0.55, 2.74)	0.619
Job status						
Unemployed, n (%)	1 (2)	13 (29)	24.38 (3.05, 194.86)	**0.003**	31.76 (3.7, 272.83)	**0.002**
Job loss**, n (%)	1 (2)	10 (22)	17.14 (2.1, 139.64)	**0.008**	31.25 (3.63, 268.83)	**0.002**
Retirement**, n (%)	1 (2)	13 (29)	24.38 (3.05, 194.86)	**0.003**	31.76 (3.7, 272.83)	**0.002**
Psychological support						
Ongoing need for psychological support, n (%)	6 (11)	14 (34)	4.06 (1.4, 11.81)	**0.01**	3.64 (1.29, 10.29)	**0.015**
Ongoing need for psychopharmacologic treatment, n (%)	2 (4)	8 (20)	6.18 (1.24, 30.93)	**0.027**	9.49 (1.9, 47.3)	**0.006**
EQ—VAS, median (IQR)	85 (80, 90)	60 (50, 75)	0.88 (0.84, 0.92)	** < 0.001**	0.88 (0.84, 0.93)	** < 0.001**

Bold values represent p-values <0.05.

*OR adjusted for age and/or sex category, respectively.

**In-comparison with pre-arrest status.

CPC, cerebral performance category; EQ-VAS, european quality of life visual analogue scale; IQR, interquartile range; OR, odds ratio.

### Secondary outcomes: health-related quality of life (EQ-5D-3L and EQ-VAS)

The mean quality of life measured by the EQ-VAS was 75.1 (17.0 SD; quartiles 65, 80, 90; range 30–100).

Key predictors for being in the lowest quartile (EQ-VAS < 65) of the EQ-VAS at 24 months of follow-up were female sex (adjusted OR 4.27, 95% CI 1.47 to 12.44; p = 0.008), ongoing need for psychopharmacological treatment at 24 months of follow-up (adjusted OR 9.16, 95% CI 2.31 to 36.33; *p* = 0.002), job-loss in comparison to pre-arrest status (adjusted OR 17.51, 95% CI 3.71 to 82.73; *p* < 0.001), retirement (adjusted OR 21.98, 95% CI 4.66 to 103.67; *p* < 0.001), evidence of PICS at 24 months of follow-up (adjusted OR 42.66, 95% CI 8.80 to 206.63; *p* < 0.001). Supplementary Fig. 1 shows the EQ-VAS results stratified by the prevalence of PICS at 24 months of follow-up.

**Figure 2:**
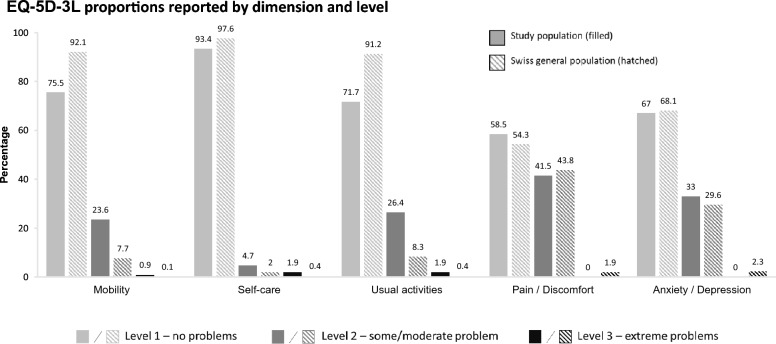
Health-related quality of Life as measured by the EuroQol 5 dimensions 3 levels questionnaire (EQ-5D-3L). EQ-5D-3L proportions are reported by dimension and level. The filled bins represent the study population, and the hatched bins represent the reference values of the Swiss general population, according to Perneger et al. Value Health. 2010.

Regarding the health-related quality of life as measured by EQ–5D–3L, most impairments were reported in the ‘pain/discomfort’ dimension, followed by the ‘anxiety/depression’ dimension and the ‘usual activities’ dimension. The least impaired dimension was the ‘self-care’ dimension. Details regarding the health-related quality of life distribution can be obtained from Fig. 2 and Supplementary Fig. 2.

## Discussion

Within this large cohort of long-term cardiac arrest survivors, more than one-third of patients had evidence of PICS 24 months after the index hospitalization. Female sex, longer no-flow time, job status at the 24-month follow-up, ongoing need for psychological/psychopharmacological treatment follow-up, lower EQ-VAS levels at the 24-month follow-up, and prevalence of PICS at the 12-month follow-up were key predictors for the prevalence of PICS. The highest burden of symptoms was observed in the physical domain, followed by the psychological domain.

The prevalence of PICS in cardiac arrest survivors might be slightly lower than in general ICU patients, as in a North American multicenter study, 56% of patients (vs. 43% in the present study) had symptoms of PICS 12 months after hospital discharge despite higher median APACHE II scores at ICU admission^9^. However, a different and performance-based cognitive assessment tool was used, which might explain the observed differences^9^.

Physical impairment, mostly resulting from muscle weakness, is a well-known long-term sequelae in survivors of critical illness, especially in sepsis and acute respiratory distress syndrome (ARDS) patients^56,57^. In a long-term study including 156 ARDS survivors, 50% of patients had persisting or resolving trajectories of muscle weakness during follow-up, associated with worse 5-year survival^56^. Also, an association between physical and psychological impairments has been described, especially in ARDS patients^58^. This is in line with our results, which also identified physical and psychological impairments as the most prevalent PICS components. However, due to our study design, we could not test for an association with mortality or identify muscle weakness as a main promotor of physical impairment.

When looking at psychological sequelae, our results are very similar to a recent Dutch study including 2′345 ICU 1-year survivors, wherein approximately 18% of unselected medical ICU patients had evidence of new depressive symptoms, 14% had evidence of new symptoms of anxiety, and 6% of patients had evidence of new symptoms of PTSD^59^. This is of great clinical relevance, as cardiac arrest survivors with symptoms of anxiety and/or depression seem to have higher long-term mortality compared to those patients without psychological problems^19^. When comparing the health-related quality of life after cardiac arrest in the 5 dimensions of the EQ-5D-3L with the reference results of the Swiss general population, the biggest differences could be observed in the ‘mobility’ and ‘usual activities’ domain, whereas in the ‘pain/discomfort’, ‘anxiety/depression’ and self-care the results were very similar to the reference results from the Swiss general population^54^. In a comparable study of 117 OHCA survivors from Norway looking at health-related quality of life after 5 years, the highest impairment was reported in the ‘mobility’ and ‘self-care’ domains of the EQ-5D-3L^60^. The mean EQ-VAS in our cohort was comparable but slightly lower than the mean EQ-VAS of the Swiss reference population^54^. This might be partially due to our cohort being older (median age 62.4 years, IQR 54.3–71.9 years) than the Swiss reference population (68% of participants were between 20 and 59 years of age), as EQ-VAS is known to have an inverse correlation with age^54^. The acceptable and comparable health-related quality of life in long-term cardiac arrest survivors is in line with a large Australian cohort study including 530 OHCA survivors 12 months after hospital discharge, where the health-related quality of life was comparable to the population norms^61^.

In the present cohort of cardiac arrest long-term survivors, the female sex could be identified as an important predictor of PICS and an EQ-VAS result in the lowest quartile. This is in line with previous research in the field, where women experience a higher prevalence of PICS and a lower health-related quality of life after cardiac arrest^21,62–64^. There is a growing body of evidence that critically ill women experience worse outcomes, especially after OHCA, even after adjusting for age and cardiac arrest circumstances^5,64,65^. Sex and/or gender differences in resource allocation, such as admission to intensive care, provision of coronary artery bypass grafting, mechanical ventilation, and targeted temperature management, were also observed in other critical care populations and might partially explain the observed differences^66–68^.

The duration of the no-flow interval as an expression of the hypoxemic stress in cardiac arrest situations was predictive for PICS even after 24 months of follow-up. This underlines the extensive damage cardiac arrest potentially causes, even in patients with good preconditions (i.e., a short no-flow time) for a favorable outcome. This corresponds with a recent multicenter study of long-term ^18-month^ outcomes in patients after OHCA, where no-flow time could be identified as a key predictor of poor functional outcomes^22^.

Job-status at long-term follow-up was identified as a further important predictor for PICS. Interestingly, having any occupational activity seems important, as retirement was also independently associated with PICS. However, the direction of the association may be two-directional, as patients with longer no-flow time and consecutive higher prevalence of PICS might experience early retirement or unemployment more frequently. In an observational cohort study of previously employed ARDS survivors, illness severity had a negative effect on return to work at 6 months^69^. Also, our measurement of neurological outcomes might not have been sensitive enough to detect subtle but debilitating long-term sequelae regarding return to work, such as delayed free recall, learning, working memory, and prospective memory^70^.

The present study has several limitations. First, there is a risk of selection bias due to the high number of patients lost to follow-up because patients who were not reachable by phone might not have been reachable due to experiencing severe psychological, physical, or neurological impairment. Alternatively, it is also possible that the patients successfully contacted were those with more severe symptomatology as patients with no or only minor symptoms might not want to be contacted anymore. However, this might be of minor concern as the included patients did not differ significantly from those lost to follow-up regarding baseline characteristics or in-hospital outcomes (Supplementary Table 2). Second, some of the observed long-term sequelae labeled as PICS might also, at least partially, be the result of hypoxic brain injury, as evidenced by the higher NSE levels in PICS patients. Third, physical impairment was measured using a self-report instrument (EQ-5D-3L), which might have yielded different results than objective tests, such as the 6-min walk test. Fourth, we used the CPC scale as a proxy for neurological outcome. Although CPC is a well-validated, expert-rated scale, its original aim was to assess overall neurological function, not specifically cognitive outcomes. Thus, there is a particular risk of underestimating the true cognitive burden in our study, as other studies have found higher proportions of cognitive impairment in the long term using performance-based assessment^34,71,72^. Fifth, as we did not assess patients' pre-arrest neurological, physical, and psychological impairments, we cannot exclude that our study overestimated the PICS burden as some patients might have had a pre-arrest history of neurological, physical, or psychological impairment. Also, the impairments observed at 24 months of follow-up might not be related to cardiac arrest. Sixth, as this is a large single-center cohort study, the presented results might not be extendable to other cardiac arrest centers, regions, or countries. Finally, we included OHCA and IHCA patients, which may differ regarding baseline factors and prognosis. However, the study sample at 24 months follow-up was too small to further look into these differences. Future multicenter studies are required to address these issues.

## Conclusions

More than one-third of cardiac arrest survivors experience symptoms of PICS within 2 years after resuscitation, with the highest impairment observed in the physical and psychological domains. However, long-term survivors of cardiac arrest report similar health-related quality of life when compared to the general population. Future studies should collect more granular data to better characterize the impact of physical/neurological/psychological impairment on return to work. Future research should also focus on appropriate PICS prevention, screening, and treatment strategies in cardiac arrest patients.

### Supplementary Information

Supplementary Figures.

Supplementary Table 1.

Supplementary Table 2.

Supplementary Table 3.

## Data Availability

The datasets generated and/or analysed during the current study are available from the corresponding author upon reasonable request.

## Abbreviations

APACHE II: Acute physiology and chronic health disease classification system II
CPC: Cerebral performance category score
CPR: Cardiopulmonary resuscitation
COPD: Chronic obstructive pulmonary disease
EQ-5D-3L: 5-Dimensions 3-levels EuroQol questionnaire
EQ-VAS: European quality of life visual analogue scale
ICU: Intensive care unit
IES-R: Impact of event scale-revised
IHCA: In-hospital cardiac arrest
IQR: Interquartile range
NSE: Neurone-specific enolase
OHCA: Out-of-hospital cardiac arrest
mRS: Modified rankin score
PEA: Pulseless electrical activity
PICS: Post-intensive care syndrome
PTSD: Post-traumatic stress disorder
ROSC: Return of spontaneous circulation
SAPS II: Simplified acute physiology score II
SD: Standard deviation
STROBE: Strengthening the reporting of observational studies in epidemiology
HADS: Hospital anxiety and depression scale
VF: Ventricular fibrillation
VT: Pulseless ventricular tachycardia
