# Supplementary material for: Post-intensive care syndrome and health-related quality of life in long-term survivors of cardiac arrest: a prospective cohort study
Source: Sci Rep. 2024 May 8;14:10533. doi: 10.1038/s41598-024-61146-8 (PMC11079009; doi:10.1038/s41598-024-61146-8)
Supplement: Supplementary file 1 — Supplementary Figures. [file 41598_2024_61146_MOESM1_ESM.pdf]

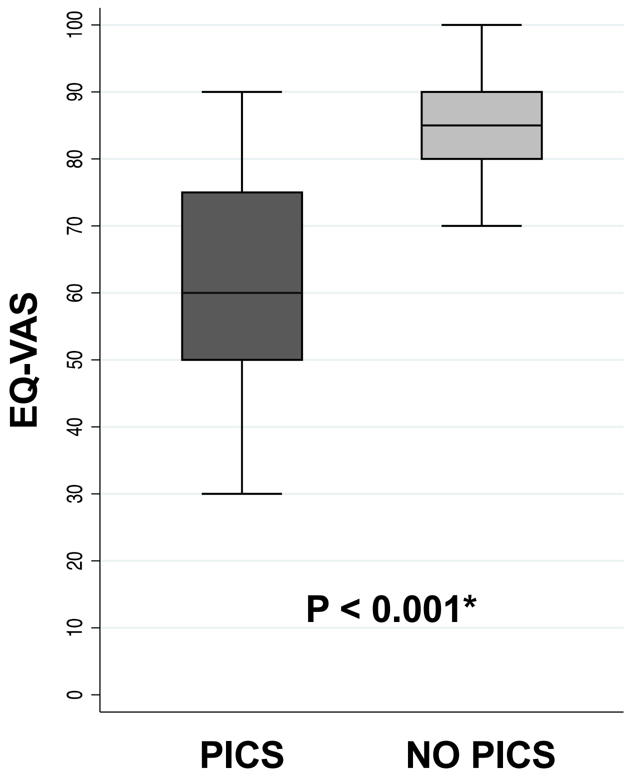

Supplementary Figure 1. Results from the EQ-VAS stratified by the prevalence of post-intensive care syndrome (PICS) at **24 months of follow-up**. The whiskers represent lowest and the highest value. The box represents 50% of the data with the 25th percentile as the lower border and the 75th percentile as the upper border. The median is depicted as a horizontal line in each box.

*P-value from the univariate model is presented.

Abbreviations: PICS *Post-intensive care syndrome.* EQ-VAS *EuroQol Visual Analog Scale.*

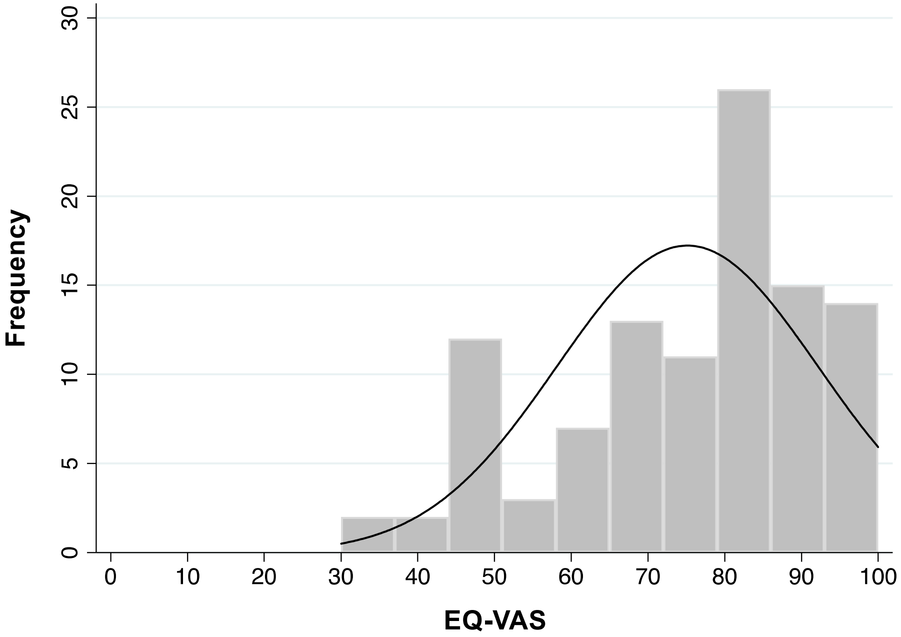

Supplementary Figure 2. Distribution of EQ-VAS results at 24-months of follow-up. *Abbreviation:* EQ-VAS *EuroQol Visual Analog Scale.*
