# Supplementary material for: Post-intensive care syndrome and health-related quality of life in long-term survivors of cardiac arrest: a prospective cohort study
Source: Sci Rep. 2024 May 8;14:10533. doi: 10.1038/s41598-024-61146-8 (PMC11079009; doi:10.1038/s41598-024-61146-8)
Supplement: Supplementary file 2 — Supplementary Table 1. [file 41598_2024_61146_MOESM2_ESM.docx]

| **Supplementary Table 1. Baseline characteristics and in-hospital outcomes stratified by out-of-hospital and in-hospital cardiac arrest.** | | | |
| --- | --- | --- | --- |
|  | **OHCA** | **IHCA** | **p- value** |
| **Sociodemographics** |  |  |  |
| N | 95 | 11 |  |
| Age (years), median  (IQR) | 62.3 (54.3, 71.9) | 63.5 (52.5, 73.9) | 0.72 |
| Female sex category, n (%) | 15 (16) | 3 (27) | 0.34 |
| Relationship, n (%) | 78 (83) | 9 (82) | 0.92 |
| Children, n (%) | 80 (84) | 7 (64) | 0.092 |
| ***Highest education*** |  |  |  |
| School, n (%) | 8 (15) | 0 (0) | 0.55 |
| Diploma/apprenticeship, n (%) | 60 (70) | 10 (100) | 0.042 |
| University, n (%) | 17 (20) | 0 (0) | 0.12 |
| Employed at baseline, n (%) | 52 (57) | 5 (45) | 0.49 |
|  |  |  |  |
| **Comorbidities** |  |  |  |
| Coronary heart disease, n (%) | 64 (67) | 9 (82) | 0.33 |
| Heart failure, n (%) | 7 (7) | 1 (9) | 0.84 |
| COPD, n (%) | 4 (4) | 1 (9) | 0.47 |
| Liver cirrhosis, n (%) | 2 (2) | 1 (9) | 0.19 |
| Arterial hypertension, n (%) | 42 (44) | 10 (91) | 0.003 |
| Diabetes, n (%) | 9 (9) | 4 (36) | 0.010 |
| Chronic kidney disease, n (%) | 4 (4) | 2 (18) | 0.058 |
| Neurological disease, n (%) | 6 (6) | 0 (0) | 0.39 |
|  |  |  |  |
| **Cardiac arrest characteristics** |  |  |  |
| ***Etiology*** |  |  |  |
| Acute coronary syndrome, n (%) | 68 (73) | 7 (64) | 0.51 |
| Rhythmogenic, n (%) | 15 (16) | 2 (18) | 0.86 |
| Other reason or unknown, n (%) | 10 (11) | 2 (18) | 0.47 |
|  |  |  |  |
| ***Setting of cardiac arrest*** |  |  |  |
| At home, n (%) | 35 (37) | 0 (0) | <0.001 |
| In public, n (%) | 60 (63) | 0 (0) |  |
| IHCA, n (%) | 0 (0) | 11 (100) |  |
| Observed cardiac arrest, n (%) | 88 (93) | 10 (91) | 0.84 |
| Bystander CPR, n (%) | 78 (82) | 8 (73) | 0.45 |
| Professional bystander CPR, n (%) | 23 (48) | 6 (86) | 0.061 |
|  |  |  |  |
| ***Initial rhythm*** |  |  |  |
| VT, n (%) | 4 (4) | 2 (18) | 0.28 |
| VF, n (%) | 74 (78) | 6 (55) |  |
| Asystolie, n (%) | 2 (2) | 0 (0) |  |
| PEA, n (%) | 5 (5) | 1 (9) |  |
| Unknown, n (%) | 10 (11) | 2 (18) |  |
| ***Resuscitation parameters*** |  |  |  |
| No-flow (min), median (IQR) | .5 (.5, 4) | .5 (.5, .5) | 0.032 |
| Low-flow (min), median (IQR) | 13.5 (10, 25) | 7 (2, 15) | 0.012 |
| Time until ROSC, median (IQR) | 16 (10, 27) | 4 (2, 15) | 0.004 |
|  |  |  |  |
| ***Epinephrine during CPR*** |  |  |  |
| No epinephrine, n (%) | 50 (57) | 4 (40) | 0.11 |
| <3 mg, n (%) | 19 (22) | 1 (10) |  |
| ≥3mg, n (%) | 18 (21) | 5 (50) |  |
|  |  |  |  |
| **Clinical scores at ICU admission** |  |  |  |
| Glasgow Coma Scale, median (IQR) | 3 (3, 8) | 15 (6, 15) | 0.001 |
| APACHE II score, median (IQR) | 28 (23, 31) | 24 (20, 26) | 0.082 |
| SAPS II score, median (IQR) | 60.5 (53, 67) | 44.5 (34, 55) | 0.019 |
|  |  |  |  |
| **ICU parameters** |  |  |  |
| pH, median (IQR) | 7.29  (7.21, 7.34) | 7.31  (7.22, 7.34) | 0.81 |
| Lactate, median (IQR) | 4.5 (2.7, 6.6) | 4 (1.2, 5.4) | 0.14 |
| Potassium (mmol/l), median (IQR) | 4.15 (3.8, 4.7) | 4.8 (4.2, 5.1) | 0.024 |
| Intubated at ICU admission, n (%) | 72 (76) | 5 (45) | 0.033 |
| Duration of invasive ventilation (days), median (IQR) | 1 (.25, 2) | 0 (0, 7) | 0.25 |
| Targeted Temperature Management, n (%) | 56 (59) | 1 (9) | 0.002 |
| Sedation, n (%) | 85 (89) | 8 (73) | 0.11 |
| NSE (ug/l) - day 2, median (IQR) | 21.6 (17.3, 29.4) | 21.1 (13.1, 25.5) | 0.29 |
| NSE (ug/l) - day 3, median (IQR) | 19.5 (16.4, 24.6) | 19.5 (13, 22.3) | 0.52 |
| ICU length of stay (days), median (IQR) | 4 (2, 7) | 3 (2, 10) | 0.61 |
|  |  |  |  |
| **ICU complications** |  |  |  |
| Aspiration, n (%) | 43 (45) | 2 (18) | 0.085 |
| Pneumonia, n (%) | 49 (52) | 1 (9) | 0.008 |
| Major hemorrhage, n (%) | 6 (6) | 1 (9) | 0.73 |
| Delirium, n (%) | 29 (31) | 3 (27) | 0.82 |
| Acute Kidney Injury, n (%) | 11 (12) | 2 (18) | 0.53 |
| Seizure, n (%) | 6 (6) | 0 (0) | 0.39 |
|  |  |  |  |
| **Hospital discharge parameters** |  |  |  |
| Hospital length of stay (days), median (IQR) | 12 (7, 16) | 10 (8, 14) | 0.82 |
| Poor neurological outcome (CPC 3-5), n (%) | 4 (4) | 0 (0) | 0.49 |
|  |  |  |  |
| **Abbreviation**s: APACHE II *Acute Physiology And Chronic Health Evaluation Score II;* CAHP *Cardiac Arrest Hospital Prognosis;* CPC *Cerebral performance category;* COPD *Chronic obstructive pulmonary disease;* CPR *Cardiopulmonary resuscitation;* ICU Intensive care unit*;* IHCA *In-hospital cardiac arrest;* IQR *interquartile range*; NSE, Neurone specific enolase; OHCA *Out-of-hospital cardiac arrest;* OR *odds ratio;* PROLOGUE *PROgnostication using LOGistic regression model for Unselected adult cardiac arrest patients in the Early stages;* ROSC *Return of spontaneous circulation* SAPS II Simplified Acute Physiology Score II; VF *Ventricular fibrillation*; VT *Pulseless ventricular tachycardia*; PEA *Pulseless electrial activity.* | | | |
|  |  |  |  |
|  |  |  |  |
|  |  |  |  |
|  |  |  |  |
|  |  |  |  |
|  |  |  |  |
|  |  |  |  |
|  |  |  |  |
|  |  |  |  |
|  |  |  |  |
