# Supplementary material for: Post-intensive care syndrome and health-related quality of life in long-term survivors of cardiac arrest: a prospective cohort study
Source: Sci Rep. 2024 May 8;14:10533. doi: 10.1038/s41598-024-61146-8 (PMC11079009; doi:10.1038/s41598-024-61146-8)
Supplement: Supplementary file 3 — Supplementary Table 2. [file 41598_2024_61146_MOESM3_ESM.docx]

| **Supplementary Table 2. Baseline characteristics and in-hospital outcomes stratified by loss of follow-up.** | | | |
| --- | --- | --- | --- |
|  | **No loss of**  **follow-up** | **Loss of**  **follow-up** | **p- value** |
| **Sociodemographics** |  |  |  |
| N | 107 | 139 |  |
| Age (years), median (IQR) | 62.4 (54.3, 71.9) | 58.6 (50, 71.7) | 0.066 |
| Female sex category, n (%) | 18 (16.8) | 26 (18.7) | 0.70 |
| Relationship, n (%) | 88 (83.0) | 100 (73.0) | 0.064 |
| Children, n (%) | 88 (82.2) | 105 (75.5) | 0.20 |
| ***Highest education*** |  |  |  |
| School, n (%) | 8 (15) | 8 (14) | 0.94 |
| Diploma/apprenticeship, n (%) | 71 (73) | 67 (74) | 0.95 |
| University, n (%) | 17 (18) | 14 (15) | 0.69 |
| Employed at baseline, n (%) | 57 (54.8) | 66 (54.1) | 0.92 |
|  |  |  |  |
| **Comorbidities** |  |  |  |
| Coronary heart disease, n (%) | 74 (69.2) | 79 (56.8) | 0.048 |
| Heart failure, n (%) | 8 (7.5) | 17 (12.2) | 0.22 |
| COPD, n (%) | 5 (4.7) | 3 (2.2) | 0.27 |
| Liver cirrhosis, n (%) | 3 (2.8) | 1 (0.7) | 0.20 |
| Arterial hypertension, n (%) | 53 (49.5) | 69 (49.6) | 0.99 |
| Diabetes, n (%) | 13 (12.1) | 21 (15.1) | 0.51 |
| Chronic kidney disease, n (%) | 6 (5.6) | 14 (10.1) | 0.20 |
| Neurological disease, n (%) | 7 (6.5) | 10 (7.2) | 0.84 |
|  |  |  |  |
| **Cardiac arrest characteristics** |  |  |  |
| ***Etiology*** |  |  |  |
| Acute coronary syndrome, n (%) | 76 (72.4) | 77 (56.6) | 0.012 |
| Rhythmogenic, n (%) | 17 (16.2) | 31 (22.8) | 0.20 |
| Other reason or unknown, n (%) | 12 (11.4) | 28 (20.6) | 0.058 |
|  |  |  |  |
| ***Setting of cardiac arrest*** |  |  |  |
| At home, n (%) | 35 (33.0) | 34 (25.2) | 0.32 |
| In public, n (%) | 60 (56.6) | 81 (60.0) |  |
| IHCA, n (%) | 11 (10.4) | 20 (14.8) |  |
| Observed cardiac arrest , n (%) | 99 (92.5) | 124 (89.2) | 0.38 |
| Bystander CPR, n (%) | 86 (80.4) | 118 (84.9) | 0.35 |
| Professional bystander CPR, n (%) | 29 (51.8) | 48 (42.5) | 0.25 |
|  |  |  |  |
| ***Initial rhytm*** |  |  |  |
| VT, n (%) | 6 (5.6) | 9 (6.5) | 0.16 |
| VF, n (%) | 80 (74.8) | 92 (66.2) |  |
| Asystolie, n (%) | 2 (1.9) | 10 (7.2) |  |
| PEA, n (%) | 6 (5.6) | 15 (10.8) |  |
| Unknown, n (%) | 13 (12.1) | 13 (9.4) |  |
| ***Resuscitation parameters*** |  |  |  |
| No-flow (min), median (IQR) | .5 (.5, 2) | .5 (.5, .5) | 0.086 |
| Low-flow (min), median (IQR) | 13 (8, 20) | 10 (6, 20) | 0.26 |
| Time until ROSC, median (IQR) | 15 (10, 26) | 13.5 (5, 21) | 0.20 |
|  |  |  |  |
| ***Epinephrine during CPR*** |  |  |  |
| No epinephrine, n (%) | 54 (55.1) | 76 (58.5) | 0.53 |
| <3 mg, n (%) | 20 (20.4) | 30 (23.1) |  |
| ≥3mg, n (%) | 24 (24.5) | 24 (18.5) |  |
|  |  |  |  |
| **Clinical scores at ICU admission** |  |  |  |
| Glasgow Coma Scale, median (IQR) |  |  |  |
| APACHE II score, median (IQR) | 27.5 (23, 31) | 27 (21, 32) | 0.74 |
| SAPS II score, median (IQR) | 60 (47, 67) | 57 (36, 66) | 0.35 |
|  |  |  |  |
| **ICU parameters** |  |  |  |
| Intubated at ICU admission, n (%) | 78 (72.9) | 98 (70.5) | 0.68 |
| Duration of invasive ventilation (days), median (IQR) | 1 (0, 2) | 0 (0, 2) | 0.028 |
| Targeted Temperature Management, n (%) |  |  |  |
| Sedation, n (%) |  |  |  |
| NSE (ug/l) - day 2, median (IQR) | 21.2 (17.3, 29) | 21.6 (17, 31.4) | 0.85 |
| NSE (ug/l) - day 3, median (IQR) | 19.4 (16.4, 23.9) | 19.1 (14.5, 28) | 0.81 |
| ICU length of stay (days), median (IQR) | 4 (2, 7) | 4 (2, 7) | 0.87 |
|  |  |  |  |
| **ICU complications** |  |  |  |
| Aspiration, n (%) | 46 (43.0) | 56 (40.3) | 0.67 |
| Pneumonia, n (%) | 51 (47.7) | 67 (48.2) | 0.93 |
| Major hemorrhage, n (%) | 7 (6.5) | 22 (15.8) | 0.025 |
| Delirium, n (%) | 33 (30.8) | 38 (27.3) | 0.55 |
| Acute Kidney Injury, n (%) | 13 (12.1) | 20 (14.4) | 0.61 |
| Seizure, n (%) | 7 (6.5) | 3 (2.2) | 0.084 |
|  |  |  |  |
| **Hospital discharge parameters** |  |  |  |
| Hospital length of stay (days), median (IQR) | 12 (8, 16) | 12 (8, 19) | 0.71 |
| Poor neurological outcome (CPC 3-5), n (%) | 1 (1, 1) | 1 (1, 1) | 0.80 |
|  |  |  |  |
| **Abbreviations**: APACHE II *Acute Physiology And Chronic Health Evaluation Score II;* CAHP *Cardiac Arrest Hospital Prognosis;* CPC *Cerebral performance category;* COPD *Chronic obstructive pulmonary disease;* CPR *Cardiopulmonary resuscitation;* ICU Intensive care unit*;* IHCA *In-hospital cardiac arrest;* IQR *interquartile range*; NSE, Neurone specific enolase; OR *odds ratio;* PROLOGUE *PROgnostication using LOGistic regression model for Unselected adult cardiac arrest patients in the Early stages;* ROSC *Return of spontaneous circulation* SAPS II Simplified Acute Physiology Score II; VF *Ventricular fibrillation*; VT *Pulseless ventricular tachycardia*; PEA *Pulseless electrial activity.* | | | |
