# Supplementary material for: Post-intensive care syndrome and health-related quality of life in long-term survivors of cardiac arrest: a prospective cohort study
Source: Sci Rep. 2024 May 8;14:10533. doi: 10.1038/s41598-024-61146-8 (PMC11079009; doi:10.1038/s41598-024-61146-8)
Supplement: Supplementary file 4 — Supplementary Table 3. [file 41598_2024_61146_MOESM4_ESM.docx]

| **Supplementary Table 3. Baseline characteristics and outcomes stratified by evidence of physical impairment in the EQ-5D-3L at 24 months follow-up.** | | | |
| --- | --- | --- | --- |
|  | **No physical impairment** | **Physical impairment** | **p- value** |
| **Sociodemographics** |  |  |  |
| N | 65 | 41 |  |
| Age (years), median (IQR) | 63.5 (57.4, 71.9) | 61.1 (52.5, 70.4) | 0.25 |
| Female sex category, n (%) | 7 (11) | 11 (27) | 0.032 |
| Relationship, n (%) | 56 (86) | 31 (78) | 0.25 |
| Children, n (%) | 55 (85) | 32 (78) | 0.39 |
| ***Highest education*** |  |  |  |
| School, n (%) | 7 (21) | 1 (5) | 0.14 |
| Diploma/apprenticeship, n (%) | 43 (70) | 27 (77) | 0.48 |
| University, n (%) | 14 (23) | 3 (9) | 0.076 |
| Employed at baseline, n (%) | 33 (52) | 23 (57) | 0.61 |
|  |  |  |  |
| **Comorbidities** |  |  |  |
| Coronary heart disease, n (%) | 46 (71) | 28 (68) | 0.79 |
| Heart failure, n (%) | 7 (11) | 1 (2) | 0.11 |
| COPD, n (%) | 2 (3) | 2 (5) | 0.64 |
| Liver cirrhosis, n (%) | 2 (3) | 0 (0) | 0.26 |
| Arterial hypertension, n (%) | 33 (51) | 20 (49) | 0.84 |
| Diabetes, n (%) | 7 (11) | 6 (15) | 0.55 |
| Chronic kidney disease, n (%) | 4 (6) | 2 (5) | 0.78 |
| Malignant disease, n (%) |  |  |  |
| Neurological disease, n (%) | 4 (6) | 3 (7) | 0.81 |
|  |  |  |  |
| **Cardiac arrest characteristics** |  |  |  |
| ***Etiology*** |  |  |  |
| Acute coronary syndrome, n (%) | 47 (75) | 29 (71) | 0.66 |
| Rhythmogenic, n (%) | 10 (16) | 7 (17) | 0.87 |
| Other reason or unknown, n (%) | 6 (10) | 5 (12) | 0.67 |
|  |  |  |  |
| ***Setting of cardiac arrest*** |  |  |  |
| At home, n (%) | 21 (33) | 14 (34) | 0.040 |
| In public, n (%) | 40 (62) | 19 (46) |  |
| IHCA, n (%) | 3 (5) | 8 (20) |  |
| Observed cardiac arrest , n (%) | 58 (89) | 41 (100) | 0.030 |
| Bystander CPR, n (%) | 53 (82) | 32 (78) | 0.66 |
| Professional bystander CPR, n (%) | 20 (53) | 9 (50) | 0.85 |
|  |  |  |  |
| ***Initial rhytm*** |  |  |  |
| VT, n (%) | 4 (6) | 2 (5) | 0.27 |
| VF, n (%) | 45 (69) | 35 (85) |  |
| Asystolie, n (%) | 2 (3) | 0 (0) |  |
| PEA, n (%) | 3 (5) | 2 (5) |  |
| Unknown, n (%) | 11 (17) | 2 (5) |  |
|  |  |  |  |
| ***Resuscitation parameters*** |  |  |  |
| No-flow (min), median (IQR) | .5 (.5, 2) | .5 (.5, 5) | 0.22 |
| Low-flow (min), median (IQR) | 12 (8, 20) | 13 (8, 30) | 0.53 |
| Time until ROSC, median (IQR) | 15 (9, 21) | 17.5 (10, 30) | 0.24 |
|  |  |  |  |
| ***Epinephrine during CPR*** |  |  |  |
| No epinehprine, n (%) | 38 (63) | 16 (43) | 0.055 |
| <3 mg, n (%) | 12 (20) | 7 (19) |  |
| ≥3mg, n (%) | 10 (17) | 14 (38) |  |
|  |  |  |  |
| **Clinical scores at ICU admission** |  |  |  |
| Glasgow Coma Scale, median (IQR) | 3 (3, 13) | 3 (3, 14) | 0.92 |
| APACHE II score, median (IQR) | 27 (23, 31) | 28 (23, 31) | 0.53 |
| SAPS II score, median (IQR) | 59.5 (42, 67) | 60 (51, 66) | 0.97 |
|  |  |  |  |
| **ICU parameters** |  |  |  |
| pH, median (IQR) | 7.28 (7.22, 7.33) | 7.31 (7.21, 7.36) | 0.38 |
| Lactate, median (IQR) | 4.25 (2.3, 6.35) | 4.5 (2.7, 6.9) | 0.79 |
| Potassium (mmol/l), median (IQR) | 4.15 (3.8, 4.8) | 4.3 (4, 4.8) | 0.34 |
| Intubated at ICU admission, n (%) | 46 (71) | 31 (76) | 0.59 |
| Duration of invasive ventilation (days), median (IQR) | 1 (0, 2) | 1 (.5, 3) | 0.31 |
| Targeted Temperature Management, n (%) | 35 (54) | 23 (56) | 0.82 |
| Mechanical circulatory support, n (%) | 1 (2) | 9 (22) | <0.001 |
| Sedation, n (%) | 56 (86) | 37 (90) | 0.53 |
| NSE (ug/l) - day 2, median (IQR) | 20.5 (17.7, 26.3) | 24.6 (16.1, 30.8) | 0.52 |
| NSE (ug/l) - day 3, median (IQR) | 19.7 (16.4, 22.4) | 19 (12.9, 33.4) | 0.96 |
| ICU length of stay (days), median (IQR) | 4 (2, 7) | 4 (2, 7) | 0.62 |
|  |  |  |  |
| **ICU complications** |  |  |  |
| Aspiration, n (%) | 31 (48) | 15 (37) | 0.26 |
| Pneumonia, n (%) | 33 (51) | 17 (41) | 0.35 |
| Major hemorrhage, n (%) | 4 (6) | 3 (7) | 0.81 |
| Delirium, n (%) | 18 (28) | 15 (37) | 0.34 |
| Acute Kidney Injury, n (%) | 5 (8) | 8 (20) | 0.071 |
| Seizure, n (%) | 3 (5) | 4 (10) | 0.30 |
|  |  |  |  |
| **Hospital discharge parameters** |  |  |  |
| Hospital length of stay (days), median (IQR) | 12 (7, 15) | 14 (9, 19) | 0.16 |
| Poor neurological outcome (CPC 3-5), n (%) | 1 (2) | 2 (5) | 0.31 |
|  |  |  |  |
| **Parameters at 24 months of follow-up** |  |  |  |
| ***Rehabilitation*** |  |  |  |
| None, n (%) | 18 (28) | 13 (32) | 0.74 |
| Up to 3 Weeks, n (%) | 22 (34) | 11 (27) |  |
| More than 3 Weeks, n (%) | 25 (38) | 17 (41) |  |
|  |  |  |  |
| ***Job-status*** |  |  |  |
| Unemployed, n (%) | 1 (2) | 13 (32) | <0.001 |
| Job loss**, n (%) | 1 (2) | 10 (24) | <0.001 |
| Retirement**, n (%) | 1 (2) | 13 (32) | <0.001 |
|  |  |  |  |
| ***Psychological support*** |  |  |  |
| Ongoing psychological support, n (%) | 7 (12) | 13 (35) | 0.008 |
| Ongoing psychopharmacologic treatment, n (%) | 3 (5) | 7 (19) | 0.036 |
|  |  |  |  |
| EQ - VAS, median (IQR) | 85 (75, 90) | 60 (50, 75) | <0.001 |
| **Abbreviations**: APACHE II *Acute Physiology And Chronic Health Evaluation Score II;* CAHP *Cardiac Arrest Hospital Prognosis;* CPC *Cerebral performance category;* COPD *Chronic obstructive pulmonary disease;* CPR *Cardiopulmonary resuscitation;* ICU Intensive care unit*;* IHCA *In-hospital cardiac arrest;* IQR *interquartile range*; NSE, Neurone specific enolase; OR *odds ratio;* PROLOGUE *PROgnostication using LOGistic regression model for Unselected adult cardiac arrest patients in the Early stages;* ROSC *Return of spontaneous circulation* SAPS II Simplified Acute Physiology Score II; VF *Ventricular fibrillation*; VT *Pulseless ventricular tachycardia*; PEA *Pulseless electrial activity.* | | | |
